# *Ce* -NeRV3D - a *C. elegans* Neuron RNA-seq Visualization tool in 3D

**DOI:** 10.17912/micropub.biology.001830

**Published:** 2025-10-24

**Authors:** Luca Golinelli, Majdulin Nabil Istiban, Jan Watteyne, Liesbet Temmerman, Isabel Beets

**Affiliations:** 1 Department of Biology, KU Leuven, Leuven, Flanders, Belgium

## Abstract

*Caenorhabditis elegans* provides a tractable model for neural circuit studies, with complete connectomes and single-cell transcriptomic atlases available. Here, we present *Ce* -NeRV3D ( *C. elegans* Neuron RNA-seq Visualization tool in 3D), a Blender plug-in that allows visualizing the expression patterns of an unlimited number of genes within the worm's 3D nervous system. The tool enables users to explore the spatial distribution of gene co-expression with customizable color mapping, overlay single-cell RNA sequencing data onto the anatomical structure, and interactively examine transcriptomic datasets in the context of detailed neural anatomy.

**Figure 1. Ce -NeRV3D is a Blender add-on module to integrate scRNA-seq data with the OpenWorm C. elegans model:**
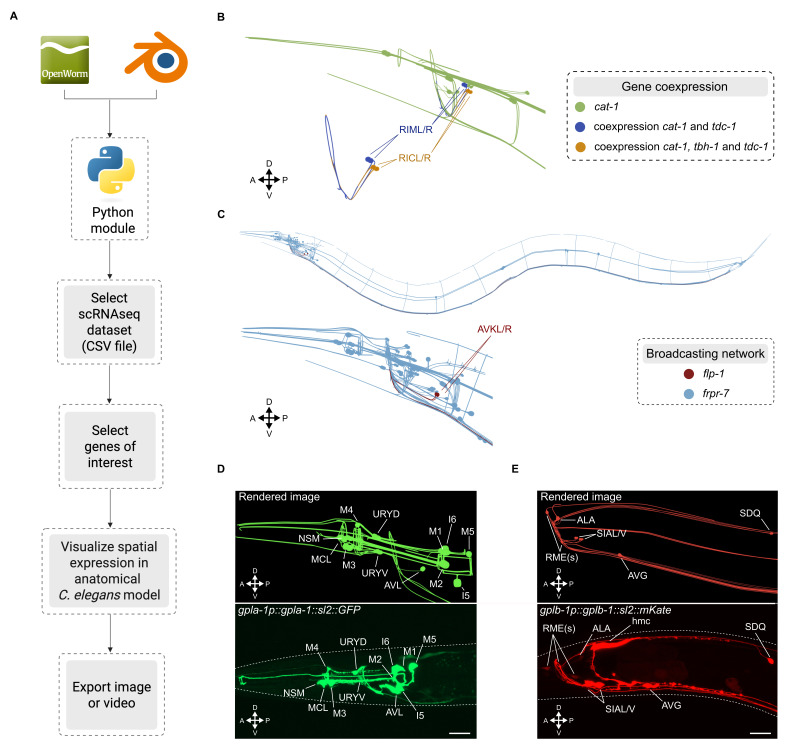
**(A)** Overview of the *Ce* -NeRV3D pipeline. **(B)** Co-expression of two genes involved in monoamine biosynthesis ( *tdc-1 , tbh-1* ) and a vesicular transporter ( *cat-1 /VMAT* ). Neurons expressing *cat-1 /VMAT* are shown in green, those co-expressing *cat-1* and *tdc-1* are shown in blue, and neurons expressing *cat-1* , *tdc-1* and *tbh-1* are shown in orange. **(C)** Visualization of a neuropeptidergic signaling network involving the neuropeptide *flp-1* , expressed in a pair of head interneurons (AVK), and its receptor *frpr-7* , which is widely expressed throughout the nervous system. Neuropeptide-expressing neurons are shown in dark red, and target neurons expressing a corresponding receptor are shown in light blue. ( **D, E)** Comparison between *Ce* -NeRV3D-rendered expression profiles and those observed in the gene's corresponding reporter strain. **(D)** Expression of *gpla-1* in the head: top part, rendered image; bottom, confocal image of a *gpla-1 ::SL2::gfp* reporter strain. **(E)** Expression of *gplb-1* in the head: top part, rendered image; bottom, confocal micrograph of a *gplb-1 ::SL2::mKate* reporter strain. ( **D, E)** Scale bar: 10 μm.

## Description

With its anatomically compact and defined nervous system, the nematode *Caenorhabditis elegans* is a powerful model for linking nervous system structure to function (Cook et al., 2019; Nguyen et al., 2016; White et al., 1986; Witvliet et al., 2021). Several single-cell RNA-sequencing (scRNA-seq) datasets are available for the *C. elegans* hermaphrodite nervous system at different life stages, from embryogenesis to adult lifespan, as well as for the sex-shared nervous system in adult males. These datasets revealed transcriptomic signatures for each neuron class and catalyzed functional studies of many genes and gene families (Cao et al., 2017; Ghaddar et al., 2023; Haque et al., 2025; Kaletsky & Murphy, 2020; Packer et al., 2019; Preston et al., 2019; Roux et al., 2023; Smith et al., 2024; Taylor et al., 2021). They also facilitated the construction of expression-based neurotransmission maps for the worm's entire nervous system, such as for monoamines and neuropeptides (Beets & Watteyne, 2025; Ripoll-Sánchez et al., 2023; Toker et al., 2025).

Most visualization tools for exploring gene expression in scRNA-seq datasets are limited to heatmap plots, omitting information about neuron morphology. The VISTA (Visualizing the Spatial Transcriptome of the *C. elegans* Nervous System) tool integrates single-cell gene expression profiles with anatomical position of neurons in the *C. elegans* nervous system (Liska et al., 2023), but this tool is restricted to the soma of each cell and shows only one gene at a time. This precludes the investigation of gene expression profiles for multiple genes within the complete neuroanatomy.

To integrate transcriptomics and anatomy, we built *Ce-* NeRV3D ( *C. elegans* Neuron RNA-seq Visualization tool in 3D) as a user-friendly tool for the spatial visualization of scRNA-seq datasets on the worm's 3D anatomy. Starting with the Virtual Worm, a publicly available 3D anatomical model of all cells within young adult *C. elegans* (Grove & Sternberg, 2011), we developed a Blender add-on that facilitates the interactive exploration of gene expression datasets within this (neuro)anatomical framework ( Figure 1A ). Blender is a free and open-source 3D modelling and rendering software that is widely used to visualize neuron morphology ( *BlenderNEURON* , 2024; Dorkenwald et al., 2024; Reinhard et al., 2024). Our tool supports any scRNA-seq dataset (in .csv format) that is structured similarly to the example file from CeNGEN (provided in the accompanying Extended Data). Once a transcriptomics dataset is uploaded, any gene can be queried in the ‘gene name' tab and the neuron(s) expressing it can be visualized using the ‘select gene' tab. This process can be repeated multiple times, as *Ce* -NeRV3D supports the simultaneous visualization of expression patterns for any number of genes. Blender also offers the ability to navigate the virtual worm in three dimensions, making it especially useful for zooming in on clusters of neurites and cell bodies. Additionally, users can identify individual neurons by simply clicking on them. Using these features, users can visualize and compare the distribution of expression for multiple genes at once, contextualized within an anatomical model of neuron morphology.

As an example, we use *Ce-* NeRV3D to visualize selected gene expression profiles of late larval (L4) stage *C. elegans* hermaphrodite neurons using the CeNGEN scRNA-seq dataset (Taylor et al., 2021). At this developmental stage, the entire nervous system has been generated, and most neurons have terminally differentiated.

We illustrate how *Ce* -NeRV3D can be used to depict neuron interactions through neuromodulatory signaling, based on expression patterns of enzymes for neuromodulator synthesis, neuropeptide-encoding genes, and receptor genes. For example, the neuromodulator tyramine (TA) is synthesized by tyrosine decarboxylase (encoded by *tdc-1* ) and subsequently converted to octopamine (OA) via tyramine-β-hydroxylase ( *tbh-1* ) (Alkema et al., 2005). While several head neurons express the vesicular monoamine transporter *cat-1* (VMAT), TA production is limited to RIM and RIC interneurons and RIC is the sole neuronal source of OA (Alkema et al., 2005; Wang et al., 2024). As shown in Figure 1B, the restricted production sites of TA and OA, with processes lying within the nerve ring, reveal a compact neuron cluster within the head neuropil for the production of these two monoamines. By visualizing the distribution of these genes' expression in conjunction with neuroanatomy, *Ce* -NeRV3D provides insights that would be difficult to discern from canonical heatmap plots for transcriptomic data alone.

Furthermore, visualizing the gene expression patterns of neuronal signaling molecules and their receptors can help contextualize cell-to-cell signaling interactions based on anatomical proximity. For example, the neuropeptide *flp-1* , expressed in AVK interneurons, forms a broadcasting network through its receptor *frpr-7* , which is expressed in many neurons across the nervous system (Ripoll-Sánchez et al., 2023). Visualization of *flp-1* and *frpr-7* expression patterns by *Ce* -NeRV3D shows that the extended morphology of AVK neurons, with processes spanning the entire length of the body, may enable short-range signaling by FLP-1 peptides to neurons located in the head, midbody, and tail ( Figure 1C ). This anatomical context can guide hypotheses on the spatial range of neural signaling by diffusible messengers, based on the anatomy of sender and receiver neurons, and can help contextualize how neuropeptides like FLP-1 may broadly impact *C. elegans '* behavior and physiology (Aoki et al., 2024; Marquina-Solis et al., 2024; Oranth et al., 2018).

We further employed *Ce-* NeRV3D to compare the reporter-based expression pattern of two peptide-encoding genes, *gpla-1* and *gplb-1* (Kenis et al., 2023), with their RNAseq-derived expression profiles ( Figure 1D- E). Taking advantage of the morphological visualization of individual neurons, our tool facilitates expression validation based on distinctive morphological features. However, the use of colocalization markers remains essential for precise neuronal identification in many instances, especially for neurons that are in proximity or share anatomical features.

The plug-in and datasets used in this study are publicly available in the Extended Data. Additional datasets not included in the repository but using the same data framework can also be used in the tool. Additionally, we provide a step-by-step protocol on how to install, generate, and export images and videos with the plug-in.

In conclusion, *Ce* -NeRV3D provides an interactive and versatile 3D visualization tool that enables the simultaneous mapping of the expression profile of multiple genes in a defined anatomical context. The tool allows for visualization of cell-resolved transcriptomics data and inference of neuronal identity based on distinctive morphological features, although the use of landmark strains remains important for confirmation. Finally, this tool provides a general methodological framework for integrating new anatomical models ( *e.g.* of different life stages or sexes), expanding its future application to developmental and sex-specific studies.

## Methods

**Collection of datasets and coding**

The 3D model of *C. elegans* was retrieved from *openworm.org* . Two scRNA-seq datasets (threshold 2 and 4) from the CeNGEN consortium were used as examples to build this tool. We scripted the code for the add-on using Python (software version 3.8.5) and tested it using Blender (software version 3.6.5). The complete code and datasets can be found in the Extended Data.

***C. elegans* maintenance**

*C. elegans* was cultured at 20°C on nematode growth medium (NGM) and fed *Escherichia* *coli* OP50 . Strains used in this study are summarized in Table 1.

**Table 1.** Strains used in this study.

**Table:** 

**Strain name**	**Genotype**	**Origin**
N2	Wild type	CGC (University of Minnesota)
IBE280	*gpla-1 ( ibt1 ) V; ibtEx43 [ gpla-1 p long:: gpla-1 gDNA::sl2::GFP:: let-858 3'UTR 40 ng/µl; unc-122p::dsRed 10 ng/µl] *	(Kenis et al., 2023)
IBE627	*gplb-1 ( ibt4 ) V; ibtEx96 [ gplb-1 p:: gplb-1 gDNA (B isoform)::sl2::mKate 40 ng/µl; unc-122p::gfp 10 ng/µl] *	This paper

**Molecular biology**

The *gplb-1 p:: gplb-1 gDNA (B isoform)::sl2::mKate* was created by PCR amplification of the *gplb-1* promoter (3589 bp upstream of the starting codon of the gene) and the B isoform of the open reading frame from N2 gDNA (mixed population). The two fragments were inserted into Gateway plasmids using standard procedures. The final plasmid was assembled via an LR recombination reaction, and its sequence was subsequently verified by sequencing.

**Generation of transgenic strains**

Young N2 adults were injected with a mix of *gplb-1 p:: gplb-1 gDNA (B isoform)::sl2::mKate* at a final concentration of 40 ng/µl, and the co-injection marker *unc-122p::gfp* at a final concentration of 10 ng/µl. Progeny of the injected worms were screened under a fluorescent stereomicroscope.

**Confocal imaging and post-analysis**

L4 animals were hand-picked on the morning of the experiment under a fluorescent stereomicroscope. Imaging slides were prepared using a 2% agar solution on glass slides. A 5 mL drop of M9 buffer was placed on the agar pad, and the worms were dispersed in the liquid. To immobilize the animals prior to imaging, a 5 µL drop of sodium azide (1 µM) was added. A coverslip was then placed over the preparation to secure it. Transgenic animals were imaged on a ZEISS LSM 900 with Airyscan 2 using 10x and 63x objectives with immersion solution. Z-stack images were captured and subsequently processed using the maximum intensity projection feature on ImageJ2 (software version 2.9.0/1.53t).

## Data Availability

Description: Ce-NeRV3D software and instructions to install. Resource Type: Software. DOI: https://doi.org/10.22002/ma8rn-yk430
